# Identification and detection of three new F17 fimbrial variants in *Escherichia coli* strains isolated from cattle

**DOI:** 10.1186/s13567-014-0076-9

**Published:** 2014-08-10

**Authors:** Morgan Bihannic, Reza Ghanbarpour, Frédéric Auvray, Laurent Cavalié, Pierre Châtre, Michèle Boury, Hubert Brugère, Jean-Yves Madec, Eric Oswald

**Affiliations:** INRA, USC1360, Toulouse, France; Inserm UMR1043/CNRS UMR5282/Université Toulouse III, Centre de physiopathologie de Toulouse Purpan (CPTP), Toulouse, France; Unité Antibiorésistance et Virulence Bactériennes, Anses Lyon, Lyon, France; Microbiology Department of Faculty of Veterinary Medicine, Shahid Bahonar University, Kerman, Iran; Unité Ecophysiologie et Détection Bactérienne, Anses Maisons-Alfort, Maisons-Alfort, France; CHU Toulouse, Hôpital Purpan, Service de bactériologie-Hygiène, Toulouse, France

## Abstract

F17 fimbriae are produced by pathogenic *Escherichia coli* involved in diarrhea and septicemia outbreaks in calves and lambs. These proteins result from the expression of four different clustered genes, namely *f17A*, *f17D*, *f17C* and *f17G*, encoding a pilin protein, a periplasmic protein, an anchor protein and an adhesin protein, respectively. Several variants of *f17A* and *f17G* genes have been reported and found genetically associated with typical virulence factors of bovine pathogenic *E. coli* strains. In this study, a new F17e-A variant, closely related to F17b-A, was identified from a collection of 58 *E. coli* isolates from diarrheic calves in Iran. While highly prevalent in Iranian F17-producing clinical isolates from calves, this variant was rare among *E. coli* from a French healthy adult bovine population, suggesting a possible association with virulence. The *f17Ae* gene was also found in the genome of the Shiga-like toxin variant Stx_1d_–producing bovine *E. coli* strain MHI813, and belonged to a gene cluster also encoding a new F17-G3 variant, which greatly differed from F17-G1 and F17-G2. This gene cluster was located on a pathogenicity island integrated in the tRNA *pheV* gene. The gene coding for a third new F17f-A variant corresponding to a combination of F17c-A and F17d-A was also identified on the pVir68 plasmid in the bovine pathogenic *E. coli* strain 6.0900. In conclusion, we identified three new F17-A and F17-G variants in cattle *E. coli*, which may also have significant impact on the development of new diagnostics and vaccination tools.

## Introduction

*Escherichia coli* is a predominant member of the normal aerobic intestinal microflora in mammals. However, due to the high genetic plasticity of the *E. coli* species, some *E. coli* strains may behave as pathogens and be responsible for a wide range of infections. These infections can be split into intestinal and extra-intestinal infections, such as urinary tract infection (UTI), meningitis or septicemia [1,2]. In veterinary medicine, different pathotypes of *E. coli* have been recognized, such as Avian Pathogenic *E. coli* (APEC) in birds or Enterotoxigenic *E. coli* (ETEC) in cattle. For pathogenic isolates, adhesion to host cells is an essential step before colonization and possible invasion, and these strains usually produce fimbrial or afimbrial adhesins to bind host cells receptors. In ruminants, some of them are involved in diarrhea (intestinal disease) or septicemia (extra-intestinal disease) outbreaks, particularly in newborn calves and lambs [3,4], and produce fimbriae of the F17 family. Despite an obvious association with virulence, the exact role of F17 fimbriae in the pathogenicity of *E. coli* remains unknown. These fimbriae were reported to bind N-acetyl-D-glucosamine (Glc-NAc)-containing receptors present on host intestinal epithelial cells in bovines [4,5]. They consist in fine filamentous heteropolymers composed of two main subunits: the structural major subunit F17-A, whose hundred copies are assembled to form the bulk of the fimbriae, and the adhesin minor subunit F17-G [4].

Among the F17 fimbriae family, several variants of the two subunits have been identified. This polymorphism was shown to result in changes in receptor specificity, in association with various virulence factors and clinical disorders [3]. Four variants of the major subunit F17-A are currently known: F17a-A, formerly named FY antigen and characterized in bovine ETEC strains [6]; F17b-A, formerly named Vir antigen and identified in *E. coli* strains isolated from septicaemic calves and lambs [7]; F17c-A, formerly called 20 K and associated with *E. coli* strains responsible for diarrhea or septicemia in calves [8]; and F17d-A, previously known as F111 and identified in bovine ETEC [9]. Two variants of the minor subunit F17-G have been identified: F17-G1 and F17-G2 [10], that are randomly associated with the 4 different variants of the subunit F17-A [11]. The F17 fimbriae are also expressed by a few human uropathogenic *E. coli* strains [12], and were described in these strains as G fimbriae [13], with two subunits GafA and GafD, that correspond to the variants F17c-A and F17-G2, respectively. Some of these variants are known to be associated with virulence factors involved in bovine pathogenesis, like CNF2 (cytotoxic necrotizing factor 2) or CDT-III (cytolethal distending toxin III) toxins [10,14,15], and the detection of these virulence factors and of F17 fimbriae is used to monitor their genetic flux and association. Two methods are widely used for the detection of F17 variants, i.e. immunological detection using specific anti-F17-A antibodies - no F17-G antibodies have been reported to date -, and PCR using either specific primers for the F17-A and F17-G variants encoding genes or consensual primers for the F17-A encoding genes family [8,10]. In F17-positive strains, negative PCR results with specific primers of every known F17 variant revealed the likely existence of unknown F17 variants [10].

In this study, we report the characterization of two new variants of the structural subunit F17-A in calves, that we propose to name F17e-A and F17f-A. A new variant of the F17-G adhesin subunit, proposed as F17-G3, was also identified, in association with the new variant F17e-A on a pathogenicity island. Based on specific primers designed in this study, we also report a very weak prevalence of F17e-A and F17-G3 in *E. coli* from healthy adult cattle. Lastly, a widely used F17-producing isolates detecting antiserum was tested on reference strains for each variant of the major subunit and was found to detect the variant F17a-A only.

## Materials and methods

### Bacterial strains and growth conditions

*E. coli* strain MHI813 (ONT:H19 serotype) was isolated from a healthy bovine feces and shown to produce the new Stx1d variant of Shiga Toxin Stx1 [16]. *E. coli* strain 6.0900 (O36:H5 serotype) was isolated from feces of a calf with diarrhea and shown to harbor the pVir68 plasmid [17]. *E. coli* strains 25KH9, S5, 31A and 111KH86 [6–9] were used as positive controls for F17a-A, F17b-A, F17c-A and F17d-A variants, respectively (Table 1). All strains were grown in Luria-Bertani (LB) broth (Invitrogen, Paisley, Scotland) for 16 h at 37 °C with 220 rpm shaking. For FY antiserum testing, *E. coli* isolates were grown overnight on polyvitaminic supplemented Minca medium (BioMérieux, Marcy l’Etoile, France) at 37 °C.

**Table 1:** **Strains used in this study**

**Bacteria**	**Strain**	**Fimbriae**	**F17-A-like subtype**	**F17-G-like subtype**	**Host**	**Pathology**	**Reference**
*E. coli*	25KH9	F17	F17a-A	F17-G1	Calf	Diarrhea	[6]
	S5		F17b-A	F17-G2	Lamb	Septicaemia	[7]
	31A		F17c-A	F17-G2	Calf	Septicaemia	[8]
	111KH86		F17d-A	F17-G1	Calf	Diarrhea	[9]
	MHI813		F17e-A	F17-G3	Cow	No path.	[16]
	6.0900		F17f-A	F17-G2	Calf	Diarrhea	[17]
	CK210	F17	F17a-A	F17-G2	Lambs and goats kids	Diarrhea	[11]
	CK377		F17c-A	F17-G2			
	CL114		F17d-A	F17-G2			
	CL394		F17d-A	F17-G1			
	IH11165		GafA	GafD	Human	Cystitis	[13]
	536	F17-related	F17-A 536	F17-G 536	Human	Cystitis	[18]
*P. mirabilis*	HI4320	Uca	UcaA	UcaG	Human	Cystitis	[19]
	HU1069		UcaA	UcaG			[20]
*B. cereus*	ATCC14579	Bcp	BcpA	BcpB	Human	Numerous	[21]

### Collection of *E. coli* isolates from diarrheic calves in Iran

Between March 2004 and June 2005, a collection of 58 *E. coli* isolates was recovered from rectal swabs of 58 diarrheic calves aged 3 to 14 days old, bred in 11 herds in the Kerman province, Southeastern Iran. The 11 herds were either in industrial or traditional rearing conditions without any history of antibiotherapy. Rectal swabs were immediately sent out to the laboratory in ice-cooled containers and streaked on MacConkey agar plates (Biolife Laboratories, Milan, Italy), which were incubated overnight at 37 °C. A single lactose-positive colony from the fecal culture from each calf was selected and confirmed biochemically to be *E. coli* (API 20E, BioMérieux, Marcy l’Etoile, France) and stored at −80 °C in LB broth with 30% sterile glycerol. After DNA extraction, virulence-associated genes were detected by PCR with specific primers of the *aap*, *aggR* and *AA probe* genes [22], *f17A* genes family, *f17Aa*, *f17Ab*, *f17Ac*, *f17Ad*, *f17G1* and *f17G2* genes [10], *afaE*-*VIII* gene [23], F5 and F41 fimbriae encoding genes [24], *stx1*, *stx2* and *eae* genes [25], *ltI*, *stI* and *ipaH* genes [26], *pap E*-*F*, *afaI* (*B*-*C*), *sfa*, *hly* and *iucD* encoding operons [27], *cnf1*, *cnf2*, *cdtIII* and *cdtIV* genes [28] and *clpG* gene [29]. The primers used and expected sizes of PCR products are presented in Table 2. The PCR products were detected by 2% agarose gel electrophoresis and 3X GelRed staining. O serogroup of *E. coli* isolates were determined with 24 antisera provided by LREC, University of Santiago de Compostela, Lugo, Spain. Phylogenetic groups (A, B1, B2, or D) were determined by triplex PCR [30] and the combinations of three genetic markers *chuA*, *yjaA*, TspE4.C2 were used for phylogenetic subgroups determination (A_0_, A_1_, B1, B2_2_, B2_3_, D_1_ and D_2_) [31]. Clonality of isolates was investigated by Pulse-field gel electrophoresis (PFGE) analysis. In brief, whole-cell DNA from *E. coli* isolates was digested with XbaI (for 16 h at 37 °C) after lysis by lysozyme and proteinase K. Electrophoresis of enzyme-generated fragments was performed with a contour-clamped electric field CHEF Mapper XA System apparatus (Bio-Rad) through a 1% agarose gel. Migration was performed for 30 h at 14 °C, with an electric field of 6 V/cm. DNA bands patterns were visualized with ethidium bromide, digitally photographed and analyzed with Gelcompar II 6.5 software. DNA bands size was determined from CHEF DNA Size Standard, Lambda Ladder (Bio-Rad) (Size range: 50–1000 kb).

**Table 2:** **Oligonucleotide primers used for detection of the virulence-associated genes on** ***E*** **.** ***coli*** **isolates from feces of calves with diarrhea in Iran**

**Gene/DNA fragment**	**Primer sequence (5′ - 3′)**	**Product size (bp)**	**Reference**
*aap*	CTTGGGTATCAGCCTGAATG	310	[22]
	AACCCATTCGGTTAGAGCAC		
*aggR*	CTAATTGTACAATCGATGTA	457	[22]
	AGAGTCCATCTCTTTGATAAG		
*AA probe*	CTGGCGAAAGACTGTATCAT	629	[22]
	CAATGTATAGAAATCCGCTGTT		
*f17A* family	GCAGAAAATTCAATTTATCCTTGG	537	[10]
	CTGATAAGCGATGGTGTAATTAAC (P2)*		
*f17Aa*	GCTGGAAGGGTGCAATACGCCTG	321	[10]
*f17Ab*	CAACTAACGGGATGTACAGTTTC	323	[10]
*f17Ac*	GCAGGAACCGCTCCCTTGGC	416	[10]
*f17Ad*	GATAGTCATAACCTTAATATTGCA	239	[10]
*f17G1*	CGGAGCTAATACTGCATCAACC	615	[10]
	TGTTGATATTCCGTTAACCGTAC		
*f17G2*	CGTGGGAAATTATCTATCAACG	615	[10]
	TGTTGATATTCCGTTAACCGTAC		
*afa E*-*VIII*	CTAACTTGCCATGCTGTGACAGTA	302	[23]
	TTATCCCCTGCGTAGTTGTGAATC		
*f5*	TATTATCTTAGG TGGTATGG	314	[24]
	GGTATCCTTTAGCAGCAGTATTTC		
*f41*	GCATCAGCGGCAGTATCT	380	[24]
	GTCCCTAGCTCAGTATTATCACCT		
*stx1*	AGAGCGATGTTACGGTTTG	388	[25]
	TTGCCCCCAGAGTGGATG		
*stx2*	TGGGTTTTTCTTCGGTATC	807	[25]
	GACATTCTGGTTGACTCTCTT		
*eaeA*	AGGCTTCGTCACAGTTG	570	[25]
	CCATCGTCACCAGAGGA		
*stI*	ATTTTTMTTTCTGTATTRTCTT	190	[26]
	CACCCGGTACARGCAGGATT		
*ltI*	GGCGACAGATTATACCGTGC	450	[26]
	CGGTCTCTATATTCCCTGTT		
*ipaH*	GTTCCTTGACCGCCTTTCCGATACCGTC	600	[26]
	GCCGGTCAGCCACCCTCTGAGAGTAC		
*papE*-*F*	GCAACAGCAACGCTGGTTGCATCAT	336	[27]
	AGAGAGAGCCACTCTTATACGGACA		
*afaI B*-*C*	GCTGGGCAGCAAACTGATAACTCTC	750	[27]
	CATCAAGCTGTTTGTTCGTCCGCCG		
*sfa*/*focDE*	CGGAGGAGTAATTACAAACCTGGCA	410	[27]
	CTCCGGAGAACTGGGTGCATCTTAC		
*hly*	AACAAGGATAAGCACTGTTCTGGC	1177	[27]
	ACCATATAAGCGGTCATTCCCGTCA		
*iucD*	TACCGGATTGTCATATGCAGACCG	602	[27]
	AATATCTTCCTCCAGTCCGGAGAAG		
*cnf1*	GGGGGAAGTACAGAAGAATTA	1111	[28]
	TTGCCGTCCACTCTCACCAGT		
*cnf2*	TATCATACGGCAGGAGGAAGCACC	1240	[28]
	GTCACAATAGACAATAATTTTCCG		
*cdtIII*	GAAAATAAATGGAATATAAATGTCCG	555	[28]
	TTTGTGTCGGTGCAGCAGGGAAAA		
*cdtIV*	CCTGATGGTTCAGGAGGCTGGTTC	350	[28]
	TTGCTGCAGAATCTATACCT		
*clpG*	GGGCGCTCTCTCCTTCAAC	402	[29]
	CGCCCTAATTGCTGGCGAC		
*chuA*	GACGAACCAACGGTCAGGAT	279	[30]
	TGCCGCCAGTACCAAAGACA		
*yjaA*	TGAAGTGTCAGGAGACGCTG	211	[30]
	ATGGAGAATGCGTTCCTCAAC		
TspE4.C2	GAGTAATGTCGGGGCATTCA	152	[30]
	CGCGCCAACAAAGTATTACG		

*P2 was used as common reverse primer for *f17A* family, *f17Aa*, *f17Ab*, *f17Ac*, *f17Ad* detection.

### In silico analysis of *E. coli* MHI813 genome and plasmid pVir68

The localization of the F17 gene cluster on the genome of *E. coli* MHI813 was determined by nucleotide basic local alignment search tool (BLASTn) analyses on *E. coli* MHI813 whole genome shotgun (wgs) sequences (taxonomy ID: 754089, GenBank:AFDZ0100000000.1) using the nucleotidic sequences of the already described F17 variants. The genetic environment of the F17 cluster on the genome was determined using BLASTn analysis of *E. coli* MHI813 wgs sequence carrying F17 cluster on the entire nucleotide collection database. All automatic annotations of coding sequences located in the vicinity of the F17 cluster were also checked with Artemis software (Wellcome Trust Sanger Institute, Hinxton, UK) and BLASTx analyses. The F17 cluster on pVir68 plasmid sequence (GenBank accession number: CP001162.1) was analyzed by BLASTn and *f17A* gene on pVir68 sequence was compared with *f17Ac* and *f17Ad* genes from 31A and 111KH86 reference strains by alignment with ClustalX2 software.

### Phylogenetic analysis of F17-A and F17-G variants encoding genes

For F17-A and F17-G phylogenetic assessment, all nucleotidic sequences of F17-A and F17-G variants were obtained from GenBank [AF022140.1, L14318.1, L14319.1, L43373.1, L43374.1, L77091.1, AFDZ01000020.1, CP001162.1, AF055306.1, AF055307.1, AF055308.1, AF055309.1, AF055310.1, AF055311.1, AF055312.1, AF055313.1, L33969.1, L43372.1, CP000247.1, U28420.1, and AM942759.1]. The sequences corresponding to the fimbrial-encoding genes *BcpA* and *BcpB* from *Bacillus cereus* strain ATCC14579 [GenBank:AE016877.1] were used as outgroups for phylogenetic analyses of *f17A* genes and *f17G* genes respectively. The sequences were aligned with MEGA 5.2 software. Based on the F17 variants alignments, phenograms were drawn using the Maximum Likelihood method based on the Tamura-Nei model [32].

### F17e-A and F17-G3 variants PCR detection

Two primers were designed with Primer3Plus software for specific PCR detection of the new variants F17e-A and F17-G3: 5′-CTGACTCCATTTACCATTGAGC-3′ for F17e-A and 5′-GCCAAATAGTGGATATGAAACAAC-3′ for F17-G3. These primers were set up in addition to the currently available multiplex PCR protocol for the detection of all known variants of F17-A and F17-G [10].

### F17e-A and F17-G3 prevalence in a healthy bovine population in France

A feces sampling campaign was performed on 787 healthy adult bovines entering in 6 slaughterhouses in Western France from October 2010 to March 2011. After enrichment in trypticase-soy novobiocine added broth overnight at 37 °C, the total DNA was extracted with the Food Extraction Pack kit (GeneSystems). A F17 screening was performed on the total DNA samples extracted from each bovine feces sample by qPCR with F17 common primers. On the F17-positive samples identified, F17e-A and F17-G3 were searched using specific primers as described above.

### FY antiserum testing on F17A variants reference strains

The FY antiserum (Biovac, Angers, France) was assessed by agglutination test on *E. coli* strains 25KH9, S5, 31A, 111KH86, MHI813 and 6.0900, which were used as reference strains for F17a-A, F17b-A, F17c-A, F17d-A, F17e-A and F17f-A variants respectively.

## Results

### A new F17e-A variant of the F17 fimbriae major subunit was detected in diarrheic calves in Iran

**Figure 1:**
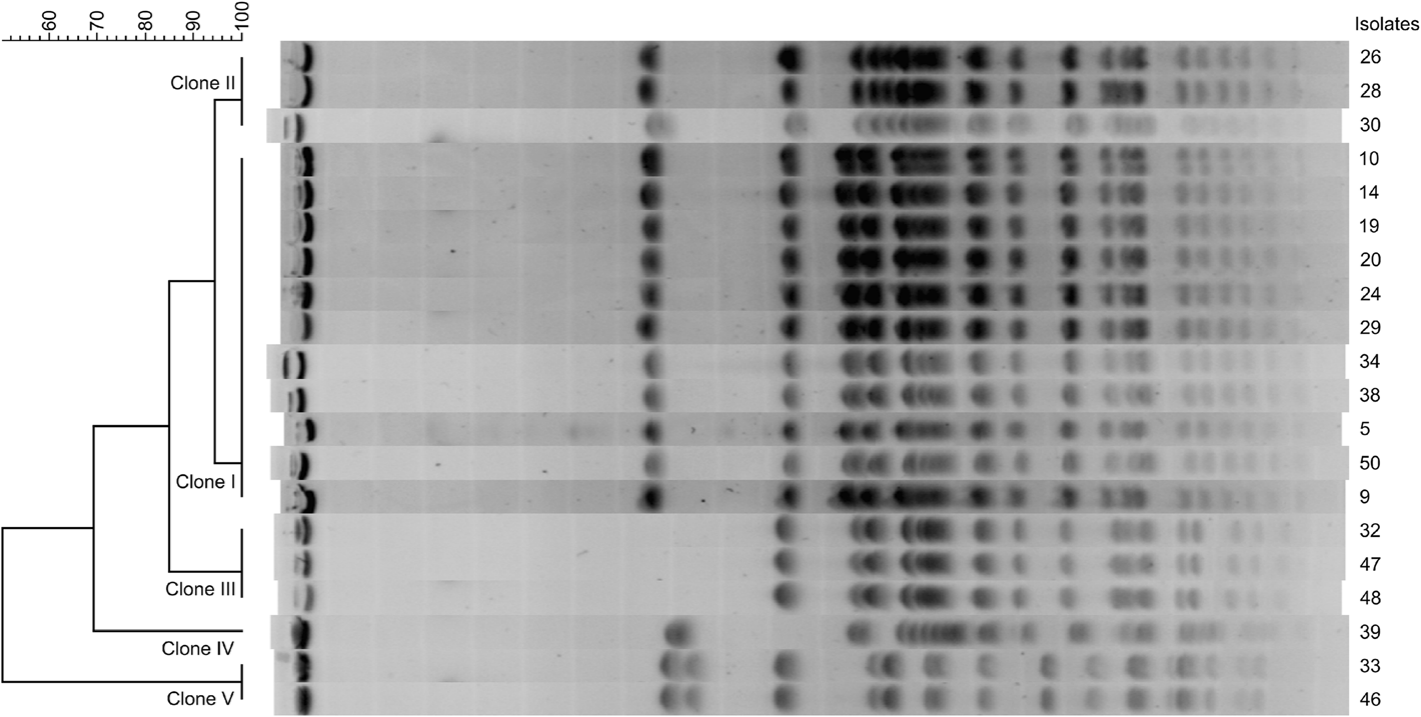
**PFGE profiles of the 20 uncharacterized F17-producing isolates of the collection of** ***E*** **.** ***coli*** **isolates from feces of diarrheic calves in Iran.** The PFGE profiles were analyzed with Gelcompar II 6.5 software, using Dice similarity coefficient, 0% optimization, 1% tolerance and UPGMA clustering method. 5 clones were identified, named from clone I to clone V.

Among the 58 *E. coli* isolates recovered from diarrheic calves in Iran, various virulence-associated genes were identified, such as the *hly*, *iucD* or *afaE*-*VIII* genes (Table 3). Interestingly, 20 out of 28 F17-A-positive isolates (71.4%), which were also shown to carry the F17-G2 variant, could not be further characterized using specific PCR for the F17-A variants. These isolates originated from 7/11 herds and corresponded to 5 *E. coli* clones (I to V), as shown by PFGE (Figure 1). Clone I and II were closely related with 90% identity on their PFGE patterns and included 11 ONT and 2 ONT and 1 O101 isolates respectively. Clone III included 3 ONT isolates, one of them carrying the enterotoxigenic *E. coli* characteristic genes *LT*-*I* and *ST*-*I*, clone IV included a single O15 isolate and clone V included 2 ONT isolates, which were found to carry the typical bovine necrotoxigenic *E. coli* genes *cnf2* and *cdtIII* [33,34]. The PCR product obtained with F17-A common primers on one *E. coli* isolate of the most prevalent clone (clone I) was sequenced and compared to the F17-A variants sequences by ClustalX2 software alignment. The F17-A PCR product sequence was found to share from 73 to 93% identity with the sequences of the four already described F17-A variants, suggesting that it was corresponding to a new variant that we named F17e-A. A specific forward primer for this new variant was designed to be used with the common reverse primer P2, which is used for detection of the *f17A* family, *f17Aa*, *f17Ab*, *f17Ac*, and *f17Ad* genes [10]. A PCR with the F17e-A specific primer was then performed on all other 19 uncharacterized F17-A *E. coli* isolates, that were found to carry the new variant F17e-A.

**Table 3:** **Detection and identification of F17 genes variants and virulence-associated genes in the collection of** ***E*** **.** ***coli*** **isolates from feces of calves with diarrhea in Iran**

**Isolate**	**Herd**	**Sampling date**	**Serogroup**	**Phylogroup**	**F17A**	**F17a-A**	**F17b-A**	**F17c-A**	**F17d-A**	**F17e-A**	**F17-G1**	**F17-G2**	**Other virulence-associated genes**
2	1	March-04	-	B1-1									
10		March-04	-	B1-1	+					+		+	
30		May-04	-	B1-1	+					+		+	
44		May-04	O8	B1-1									
38		May-04	-	B1-1	+					+		+	
49		June-04	-	B1-1									
52		June-04	O8	B1-1									
64		March-05	O157	A-1									*afaE*-*VIII*, *iucD*
16	2	April-04	-	B1-1									
68		April-05	-	B1-1	+			+				+	*iucD*
73		June-05	-	D-1									*afaE*-*VIII*, *clpG*, *iucD*
45	3	May-04	-	B1-1									
57		June-04	O15	B1-1	+		+				+		
4	4	March-04	O8	B1-1									
3		March-04	O8	B1-1									
29		April-04	-	B1-1	+					+		+	
56		June-04	O8	A-1									*afaE*-*VIII*, *iucD*
25	5	April-04	-	B1-1	+	+						+	
35		May-04	-	B1-1	+	+						+	
63		March-05	O49	B1-1									
5	6	March-04	-	B1-1	+					+		+	
26		April-04	-	B1-1	+					+		+	
28		April-04	O101	B1-1	+					+		+	
32		May-04	-	B1-1	+					+		+	
51		June-04	O141	B1-1									
54		June-04	-	B1-1									
9	7	March-04	-	B1-1	+					+		+	
11		March-04	O8	B1-1	+			+				+	*iucD*
14		March-04	-	B1-1	+					+		+	
46		May-04	-	B1-1	+					+		+	*cnf2*, *cdtIII*
43		May-04	O8	B1-1									
33		May-04	-	B1-1	+					+		+	*cnf2*, *cdtIII*
66		March-05	O8	A-2									
69		April-05	O8	A-2									
75		June-05	O8	B1-1	+			+				+	*iucD*
74		June-05	O8	B1-1	+			+				+	
19	8	April-04	-	B1-1	+					+		+	
34		May-04	-	B1-1	+					+		+	
47		May-04	-	B1-1	+					+		+	
48		June-04	-	B1-1	+					+		+	*stI*, *ltI*
53		June-04	-	A-1									*iucD*
59		June-04	-	B1-1									
62		March-05	-	B1-1									
65		March-05	-	D-1									*hly*
71		April-05	O8	B1-1									
39	9	May-04	O15	B1-1	+					+		+	
31		May-04	-	B1-1									
60		June-04	O49	B1-1									
70		April-05	-	A-1									
27	10	April-04	O8	B1-1									
20		April-04	-	B1-1	+					+		+	
24		April-04	-	B1-1	+					+		+	
37		May-04	O8	B1-1									
41		May-04	O8	B1-1									
50		June-04	-	B1-1	+					+		+	
72		April-05	O15	B1-1	+				+		+		
55	11	June-04	-	D-1									*stI*, *ltI*
61		June-04	-	A-2									*hly*

### The F17e-A variant is encoded on a gene cluster carried by a pathogenicity island on *E. coli* MHI813 genome, in association with a new variant F17-G3 of the minor subunit

**Figure 2:**
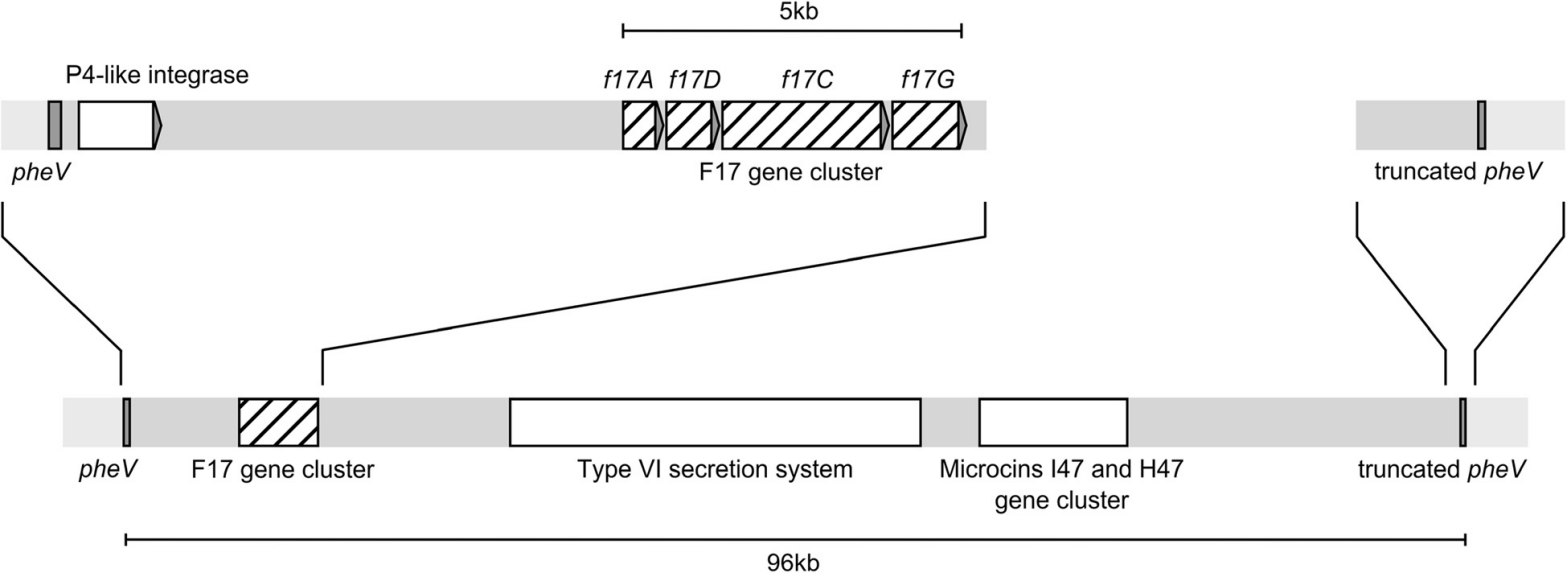
**Map of the** ***E*** **.** ***coli*** **strain MHI813 pathogenicity or fitness island carrying the F17e-A and F17-G3 variants gene cluster.** This 96 kb genomic island is inserted into the phenylalanine tRNA encoding gene *pheV* and carries notably a P4–like integrase gene, close to the insertion site, F17 fimbriae encoding genes, organized in a gene cluster, a type VI secretion system gene cluster and a microcins I47 and H47 gene cluster.

The MHI813 strain was previously detected to be positive with F17-A common primers PCR but tested negative with F17a-A, F17b-A, F17c-A, and F17d-A specific PCRs as well as with F17-G1 and F17-G2 specific PCRs (data not shown). However, the MHI813 strain was now found positive using the newly designed F17e-A specific primer. To identify the localization and the genetic environment of the *f17Ae* gene on MHI813 genome, the wgs sequence of MHI813 strain was used. The in silico analysis of MHI813 genome revealed that the F17 fimbriae encoding genes, ie *f17A*, *f17D*, *f17C* and *f17G*, were organized in a gene cluster, which was identified on the contig 19 of MHI813 strain wgs sequence. The N-terminal half of the adhesin encoding gene *f17G* on MHI813 genome also showed no identity with the same part of the adhesin genes of the other variants. This result suggests the expression of a very different adhesin in MHI813 strain, that we named F17-G3, and for which we developed a specific primer for its detection by PCR. The nucleotide sequences of *f17Ae* and *f17G3* genes located on MHI813 genome are presented in Table 4. The F17 cluster was otherwise found to be carried by a genomic island, that straddles the contigs 19 and 20 of MHI813 wgs sequences. This genomic island was shown to be inserted in the tRNA *pheV* gene. Other putative genes were found as well, notably a P4-like integrase gene close to *pheV* gene, and gene clusters and operons like type VI secretion system gene cluster, microcins I47 and H47 gene cluster (Figure 2), suggesting that it actually constitutes a pathogenicity or fitness island.

**Table 4:** **Nucleotide sequences of the new F17 variants genes** ***f17Ae*** **,** ***f17fAf*** **and** ***f17G3***

**Variant gene**	**Nucleotide sequence**	**GenBank accession number (nucleotides)**
*f17Ae*	ATGCGGAAAATTCAATTTATCCTTGGAATACTGGCGGCTGCGTCATCTTCTGCTACGCTTGCTTATGACGGTACAATTACTTTTAATGGAAAAGTTGTTGCTCAGACTTGTTCTGTTTCATCAGGAAGCAAGAATTTAACCGTTACATTACCGACTGTTTCTGAAGCTTCATTGGCTGCCGCAACAAATACTGCAGGTCTGACTCCATTTACCATTGAGCTGACCGGGTGTGATACCAATGCAGCTTCTGGTGCTCAGAACGTAAAAGCTTATTTTGAACCTAACGCCACGACTGATTACGATACGGGTAATCTAAATATTGCTGCCAGTGGTGCAAATAACGTTCAGATACAGCTTCTAAATGCAGATGGAGTTACTCCAATAAAACTAGGCCAAGATGCTACAGGACAGAATGTTACAGCTGTACAGATTGATAACGCAGCTATGAAACTGCGTTATAATGCACAATATTATGCCACAGCTCAGGCTACAGCTGGTGATGTCTCTGCCACAGTAAATTACACCATCGCGTATCAGTAA	AFDZ01000020.1 (420293-420832)
*f17Af*	ATGCAGAAAATTCAATTTATCCTTGGAATACTGGCGGCTGCGTCATCTTCTGCTACGCTTGCTTATGACGGTACAATTAATTTTACTGGGAAAGTCGTTGATCAAACTTGTTCTGTTACTACAGGAACCGCTCCCTTGGCAGTTACACTACCAACTGTCTCCACAAAATCATTAGATTCAACCGGGAAGGTAGCAGGCCTTACTCCTTTCACAATTTCACTGAGTGGGTGTAATACTGCTGCCGCTACAGGAGCTCAAAGTGTGAAGGCTTATTTTGAGCCTAATGCGACCACTGATTATGATAGCCATAACCTTAATATTTCAAGTAGTGGCTCTGGTAACGCAACAAATGTCCAGATTCAGCTTCTGAATGCTGATGGTACCACACAGATACTACTTGGTAATGATTCTGCTACTCAGGGAGTAACACCTGTTGTTATTAATAATGACGCAATGACATTGCGTTATAACGCACGGTACTATGCAACAGGTCAGGCCACCGCCGGCAACGTTTCTGCCACTGTGAATTACACCATCGCTTATCAGTAA	CP001162.1 (9749-10297)
*f17G3*	ATGATTTTCAGATATATCAGATTATTTTTAGTTGTACTTTGCAGTGTTGTTCTGGTTGACAGGGGAAACGCAGCATCTTTGTTTAATATCACGTTTGTCGGTAATGCTGAACAAACAATCACACCATCATCTGCATATACACTGACGCATGCAATGGATAATCTGCCTTACGTGTTCGATGAGGCGGGAGTAACTATTGGTTATTCCGCGGTTACTGTCTGGCAATATCCACGAGGGGTAAGAGTGTGTGCCGGGCTTGATGCAAAAGTTGGTTTACCCGTAGTGGGTAGCATTAATGGTCAGGATATCTATGGCGTCACTGATGAAGTGGGGCTGCTGGTATGGATGGGGGATGCCGGATATTCAGATGATGTTGCAATGACCGGAAATACGTGGACAAATGTACTGGATTCATGGTGTACAGCAAACGTTCCTCCAACGAGCCAGGGACTATCCCTTTATGTAAAACCTGTCATTCTTAAAAGAAGTACTACTGCTTCTTATGTTATCCCTCAGACGACAATAGGTAGTATTAAGTTTCGTCTGGAAGATGGGCCAAATAGTGGATATGAAACAACTGTCAATTTTACACTCAGTAGTTTTACGATTAACAATACAGTCACATCGTGCAGACTGTTGACGCCGGCATCCGTCAATGTTGCATTGCAGGACGTTTTTGTCAGTCAGTTTCCCTCTTCCGGTGATGAGGTTGCAGCCGGCTCCACGACGTTGCGGCTGCAGTGTGATGCAGGAGTGACGGTATGGGCAACACTGACTGATGCGACCACACCGTCCAACAGAAGCGATATACTCACACTGACGGGGGCATCGACTGCAACCGGAGTCGGGCTGAGAATATACAAGAACACTGACAGTTTGCCCCTGAAGTTTGGACCTGATTCGCCGGTAAAGGGAAATGAAAACCAGTGGCAGTTATCGTCAGGGACGGAAACGTTGCCCTCAGTTACCCTGAATGTAAAGTATGTTAATACCGGTGAGGGAATTAATCCGGGTACGGTCAACGGAATATCAACATTTACGTTTTCCTATCAGTAA	AFDZ01000020.1 (424180-425235)

### The F17e-A and F17-G3 prevalence in healthy bovines in France is very low

To further assess the genetic flux of these two new variants F17e-A and F17-G3, we investigated their prevalence in faecal samples from 787 healthy adult cattle in 6 slaughterhouses in Western France. Among the 787 samples analyzed, 373 were found positive for F17 by qPCR. On the 373 F17-positive samples, 3 were shown to be positive for F17e-A (3/373, 0.8%). No F17-G3 positive sample was detected.

### Plasmid pVir68 carries a new variant F17f-A, which corresponds to a combination of F17c-A and F17d-A

**Figure 3:**
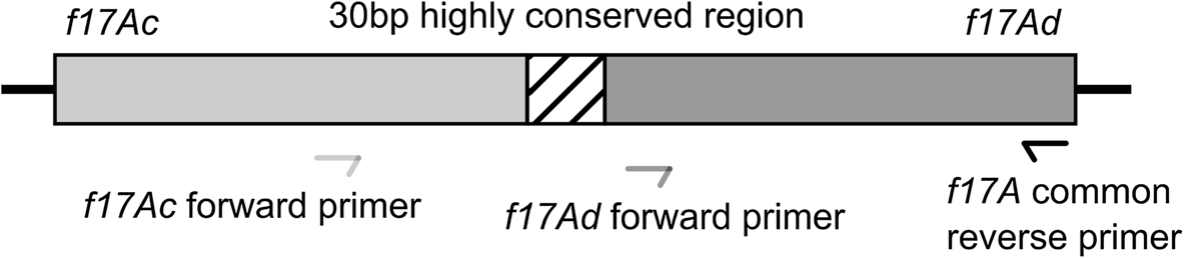
**Map of the** ***f17A*** **gene carried on plasmid pVir68.** This gene corresponds to a mixture of *f17Ac* and *f17Ad* genes. The highly conserved region identified is shared by the both variants genes *f17Ac* and *f17Ad* and constitutes a likely recombination region. This region is located between the two matching regions of the forward specific primers for *f17Ac* and *f17Ad*.

The 6.0900 strain was previously detected positive by PCR with F17-A common primers, and with F17c-A and F17d-A specific primers (data not shown). The 6.0900 strain plasmid pVir68, which was sequenced by Johnson et al. [17], is known to carry a F17 fimbriae encoding gene cluster. To clarify the F17-A status of 6.0900 strain, an in silico analysis of the plasmid pVir68 sequence was undertaken. It showed that the *f17A* encoding gene on pVir68 corresponds to a combination of F17c-A and F17d-A variants, with the N-terminal part corresponding to the same part on *f17Ac* gene and the C-terminal part corresponding to the same part on *f17Ad* gene (Figure 3). A 30 bp highly conserved area was also identified between the *f17Ac* gene corresponding N-terminal part and the *f17Ad* gene corresponding C-terminal (264–292 bp area on plasmid pVir68 *f17A* gene). Sequencing of F17c-A specific PCR product obtained on 6.0900 strain was then performed, and the in silico analysis of the genetic sequence of the PCR product confirmed that the *f17A* gene of pVir68 was a combination of *f17Ac* and *f17Ad* genes. We proposed F17f-A for this new variant of the major subunit. f*17Af* nucleotide sequence is presented in Table 4.

### F17e-A and F17-G3 phylogenetically diverge from the other variants of F17-A and F17-G

**Figure 4:**
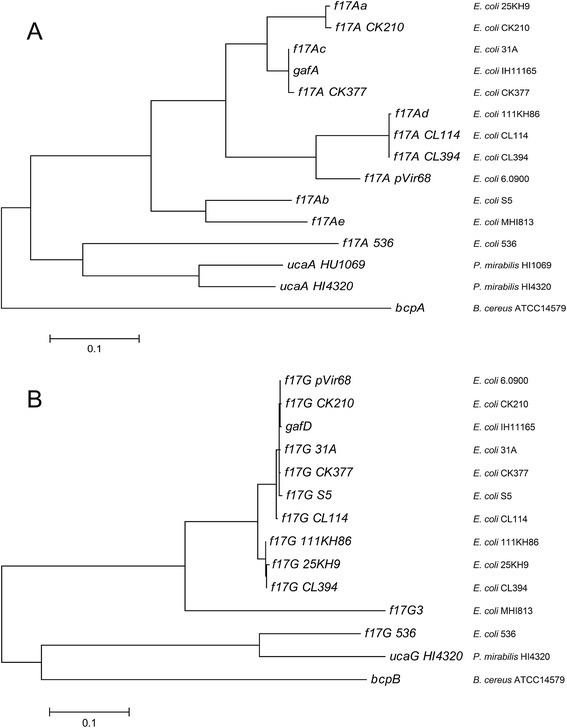
**Phenograms of F17-A variants and F17-A-related genetic sequences (A) and F17-G variants and F17-G-related genetic sequences (B).** The phenograms were drawn using the Maximum Likelihood method based on the Tamura-Nei model [32]. The trees with the highest log likelihood (−3962.3380 for F17-A, and −4843.1422 for F17-G) were shown. The trees are drawn to scale, with branch lengths measured in the number of substitutions per site.

In order to clarify the phylogenetic links between the 4 F17-A and 2 F17-G variants reported up to now and the F17e-A, F17f-A and F17-G3 newly described, a phylogenetic analysis was performed with the *f17A* and *f17G* gene sequences from several *E. coli* F17-producing strains and *E. coli* and *P. mirabilis* F17-related producing strains (Table 1). In the two phenograms, all gene sequences encoding a variant were observed in related leaves (Figure 4). *f17Ae* gene sequence was found to be related to *f17Ab* gene sequence. On the other hand, *f17G3* gene was found to significantly diverge from the genes coding the two variants F17-G1 and F17-G2.

### The FY antiserum detects F17a-A variant-producing isolates only

The FY antiserum is commonly used in veterinary laboratories to detect all F17-producing *E. coli* isolates involved in diarrhea or septicemia outbreak in calves. To check the ability of this antiserum to detect all F17-A variants, we performed agglutination testing using the appropriate reference strains for each F17-A variant, including the MHI813 and 6.0900 strains for the new variants F17e-A and F17f-A. A positive agglutination reaction was only observed with the 25KH9 reference strain only, suggesting that this antiserum is unable to detect other F17a-A variants.

## Discussion

In this study, we report three new variants of the pilin and adhesin subunits F17A and F17G of the F17 fimbriae in *E. coli*. The F17e-A variant was identified in *E. coli* isolates recovered from feces of diarrheic calves in Southeastern Iran. The prevalence of this variant among the F17-A positive *E. coli* isolates (71.4%) was surprisingly high in this cattle population. These F17e-A isolates were identified in 7/11 herds, suggesting a wide spread of the variant F17e-A in the Kerman province. These isolates were found in 5 *E. coli* clones, three of them harbouring the F17e-A variant only. Moreover, the *cnf2* and *cdtIII* genes were found in association with F17e-A in a fourth clone. Interestingly, the most prevalent clone I and the 3 clones II, III and V were detected in the same herds suggesting the presence of a major clone disseminating the F17e-A variant. Considering that a plasmidic location of the *f17Ae* gene would probably be associated with a higher clonal diversity of the *f17Ae*-carrying *E. coli* thanks to intercellular plasmid spread, the hypothesis of a chromosomal location of *f17Ae* can be put forward. However, this would need further molecular confirmation.

In three clones (I, II and IV), the F17e-A variant was the unique virulence-associated factor identified. Considering that those calves were severely affected with diarrhea, this may suggest the expression of a still unknown virulence factor associated to the F17e-A variant. Therefore, it could be of interesting value to further determine the whole sequence genome of these *E. coli* clones in order to detect in silico possible new virulence factors involved in diarrhea outbreak in calves.

The new variant F17e-A was also identified in the *E. coli* MHI813 strain, which was isolated from bovine feces. It was found to be associated on the MHI813 strain genome to a new variant of the adhesin subunit, F17-G3. These two variants were found into a gene cluster, confirming the previous observations of a co-localization of the F17 fimbriae encoding genes in a cluster organization [10,35]. Moreover, the co-localization of a type VI secretion gene cluster, and a microcins I47 and H47 gene cluster is reminiscent of a pathogenicity or fitness island [36,37]. The integration of this genomic island within the *pheV* gene, which encodes phenylalanine tRNA, also supports this hypothesis. Indeed, the *pheV* gene is localized at the 67^th^ minute on the *E. coli* K12 genome map, which is a known hot spot for pathogenicity island insertions [38-41].

The new variant F17-G3 was found to share no identity on its N-terminal encoding part with the two already reported variants F17-G1 and F17-G2, what was highlighted by the great phylogenetic divergence of F17-G3 with the two others adhesin variants. The N-terminal part of the adhesin is known to be responsible for the adhesion onto the host receptor [4] and the completely different N-term part of F17-G3 may indicate some differences in receptor specificity or even in host tropism. Therefore, a functional characterization of this new variant may help investigating the F17-G3 adhesion properties. Concerning the two variants F17-G1 and F17-G2, the phylogenetic analysis showed a very close relatedness. These two variants share 99% identity, and we can wonder whether the weak genetic differences between these two variants do really correspond to biological differences, e.g. in terms of adhesion specificity.

Regarding the genetic flux of the two new variants F17e-A and F17-G3, the prevalence of the variant F17e-A was very low in healthy adult bovines in France. F17-G3 was even not detected at all in these animals. One hypothesis would rely on a very low prevalence in cattle, those variants being incidentally identified in a restricted area (Iran) during a short period of time. Another hypothesis could be that these two variants are strongly associated with virulence in calves, as it is the case for the F17b-A variant, which is known to be mainly associated to CNF2 producing *E. coli* strains, both of them being carried by the Vir plasmid [4,10,14].

Another new variant F17f-A of the major subunit was identified on the recently sequenced virulence plasmid pVir68, that carries different virulence factors encoding genes, like *cnf2* and *cdtIII* genes [17]. This new variant corresponds to a combination of the already described variants F17c-A and F17d-A. The structure of this new variant suggests that the highly conserved regions previously identified [4] on the different variants of the pilin and adhesin subunits could be the target of recombination events, which would constitute a potential source of genetic diversity. This strategy may allow the F17-producing strains to bypass the immune defense system of the host, particularly the specific antibodies developed against the major subunits variants of F17 fimbriae after a first contact with a F17-producing pathogenic *E. coli*.

From all new variants and the already reported ones for the major subunit of F17 fimbriae, and as tested through agglutination reactions, the FY antiserum of Biovac was able to detect the F17a-A variant only. This does not exclude that the FY antiserum would have detected other variants using different approaches, such as immunoblotting. However, our point was to assess the capabilities of this serum to detect F17-producing *E. coli* from diarrheic calves in the same conditions as routinely performed by veterinary laboratories. Further work is required to provide new immunological tools for the detection of all F17 variants for routine purposes. Interestingly, the same F17a-A variant is used to produce the two vaccine formulas of Trivacton6® and Imocolibov® purchased by Merial company. Accordingly, serious doubts on the ability of these vaccine formulas to confer a total protection against all the F17-producing *E. coli* strains can be put forward. As noticed above for immunological reagents, it would be interesting to consider all the described variants of the major subunit for their inclusion in these vaccines.
